# CAX1 beyond calcium transport: evidence for a transport-independent regulatory module in plants

**DOI:** 10.3389/fpls.2026.1937940

**Published:** 2026-09-07

**Authors:** Fernando Chavira, Daniella Chacon-Avila, Shayan Sarkar, Kendal D. Hirschi, Ricardo Andres Bernal

**Affiliations:** 1 Department of Chemistry and Biochemistry, University of Texas at El Paso, El Paso, TX, United States; 2 Department of Biological Sciences, University of Texas at El Paso, El Paso, TX, United States

**Keywords:** amino-terminal regulatory region (NRR), Ca^2+^ Cation Antiporter (CaCA), Ca^2+^/H^+^ antiport, calcium homeostasis, Cation/H^+^ exchanger 1 (CAX1), kinase-dependent phosphorylation, plant calcium signaling, protein interactions

## Abstract

Plant cation/H+ exchanger 1 (CAX1) is a vacuolar Ca^2+^/H^+^ antiporter that plays a central role in calcium homeostasis. The initial 36 residues of CAX1 make up the amino-terminal regulatory region (NRR), this defining feature mediates autoinhibition and serves as a target for phosphorylation and protein-protein interactions. While CAX1 has been viewed as a transporter, recent genetic studies suggest a broader functional role. Loss of CAX1 enhances tolerance to anoxia and submergence stress, while synthetic biology reveals that transport-deficient amino-terminal modules of CAX1 influence stress responses. Notably, a dominant-negative amino-terminal construct phenocopies *cax1* loss-of-function mutants, whereas a transport-deficient amino-terminal module lacking the autoinhibitory region restores anoxia sensitivity in a *cax1* background. These observations are difficult to explain through altered calcium transport alone. In this review, we summarize current understanding of CAX1 structure, regulation, and physiological function and evaluate evidence supporting transport-independent activities of the amino terminus. We propose that the amino-terminal architecture of CAX1 could function as a control module that integrates environmental and cellular cues to influence stress responses. This framework expands the functional scope of CAX1 beyond ion exchange and might suggest that membrane transport proteins contribute directly to signaling networks.

## 1 Introduction

Calcium (Ca^2+^) is one of the most versatile signaling molecules in plants, participating in processes ranging from growth and development to responses against abiotic and biotic stress (Pittman and Hirschi, 2024; Wdowiak et al., 2024; Luan, 2026). Environmental stimuli including salinity, drought, temperature extremes, flooding, and pathogen attack trigger transient changes in cytosolic Ca^2+^ concentration that are decoded by diverse downstream signaling pathways (Aldon et al., 2018; Bakshi and Gilroy, 2024; Wang et al., 2024). The amplitude, duration, and spatial characteristics of these calcium signatures determine the specificity of cellular responses (Luan, 2026). Consequently, mechanisms that regulate intracellular calcium homeostasis are central components of plant signaling networks (Cai and Lytton, 2004; Shigaki and Hirschi, 2006; Wdowiak et al., 2024).

**Figure 1:**
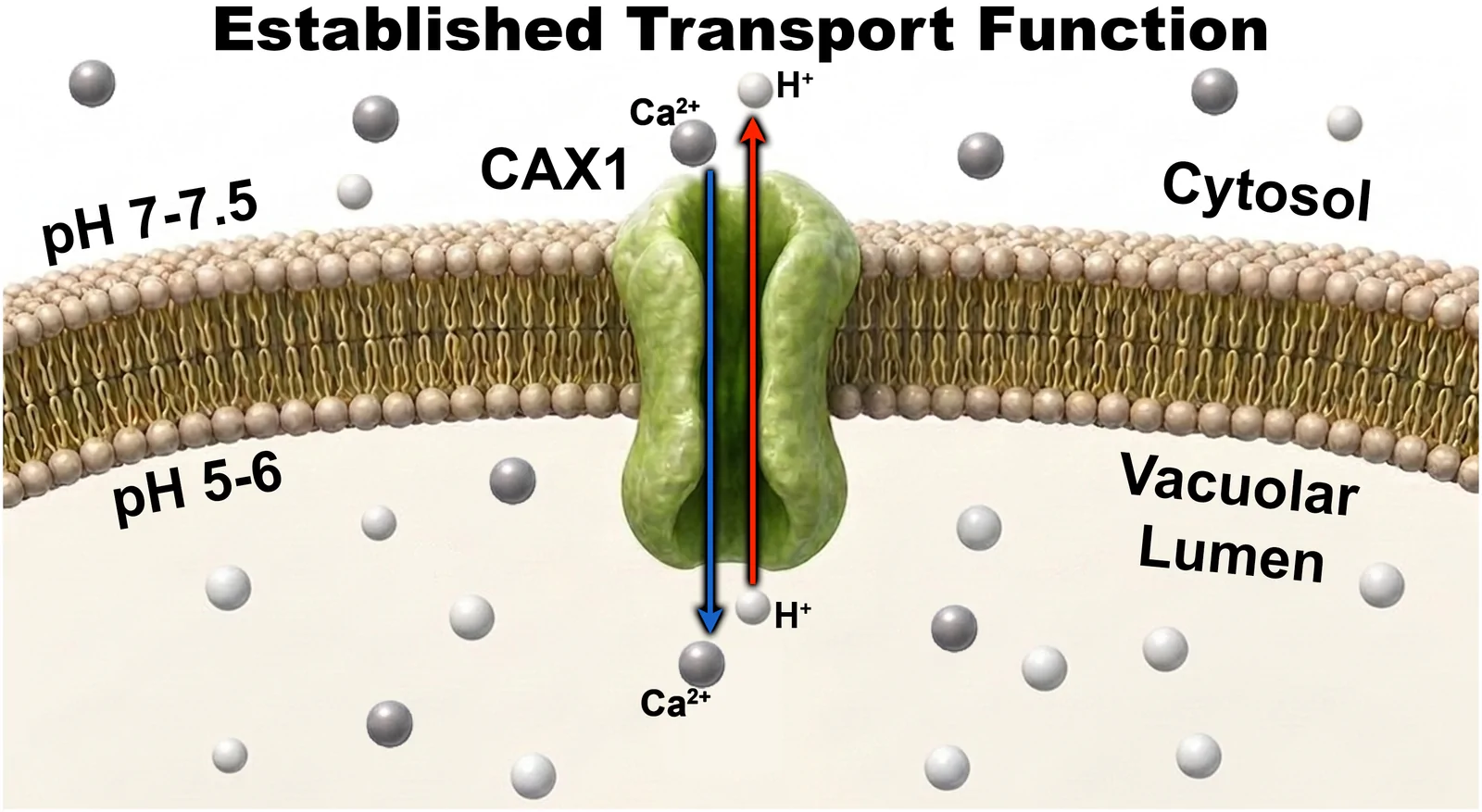
Established Ca^2+^/H^+^ antiport function of CAX1. Schematic representation of the canonical transport activity of CAX1 at the vacuolar membrane. CAX1 functions as a Ca^2+^/H^+^ antiporter that uses the proton gradient across the tonoplast to drive Ca^2+^ sequestration from the cytosol into the vacuolar lumen. The cytosolic side is shown at near-neutral pH, approximately pH 7–7.5, whereas the vacuolar lumen is acidic, approximately pH 5–6. The downward Ca^2+^ arrow indicates Ca^2+^ transport into the vacuole, while the upward H^+^ arrow represents proton counter-transport from the vacuolar lumen toward the cytosol. This model summarizes the established transport-centered function of CAX1 in vacuolar Ca^2+^ homeostasis.

Among these mechanisms, vacuolar calcium transport plays a particularly important role (Conn et al., 2011; Demidchik et al., 2018). The vacuole represents the largest intracellular calcium reservoir in plant cells and serves as a major determinant of cytosolic calcium buffering capacity (Conn et al., 2011). Calcium movement across the tonoplast is controlled by coordinated activities of calcium pumps and calcium/proton exchangers that together regulate calcium storage, mobilization, and signaling competence (Conn et al., 2011; Pittman et al., 2011). Members of the cation/H^+^ exchanger (CAX) family are key components of this system, using the proton motive force generated by vacuolar proton pumps to drive calcium accumulation within the vacuole (Hirschi et al., 1996; Shigaki and Hirschi, 2006) (**Figure 1**). Through this activity, CAX transporters influence calcium allocation, mineral nutrition, development, and environmental stress responses (Bakshi et al., 2023; Pittman and Hirschi, 2024).

Among the six *Arabidopsis* CAX proteins, CAX1 has emerged as one of the most extensively studied members of the family (Pittman and Hirschi, 2016a; Mao et al., 2021). Early work established CAX1 as a vacuolar Ca^2+^/H^+^ antiporter and identified a distinctive NRR that suppresses transporter activity (Hirschi et al., 1996; Pittman and Hirschi, 2001). Although notable for its capacity to generate a constitutively active transporter, removal of the CAX1 NRR should not be regarded simply as an artificial manipulation lacking physiological relevance (Pittman et al., 2002b; Pittman et al., 2002a). Indeed, evidence from *Chlamydomonas reinhardtii* indicates that naturally occurring CAX transcript variation can generate forms differing in their amino-terminal regions (Pittman et al., 2009). Moreover, experimental truncation of the CrCAX1 amino-terminus selectively enhanced Ca^2+^/H^+^ exchange without similarly affecting Na^+^/H^+^ exchange, demonstrating that this region can differentially regulate distinct CAX activities. Thus, truncated CAX constructs provide both a means of probing the functional architecture of CAX proteins and a potential model for physiologically relevant variation in amino-terminal regulation. Subsequent studies have identified multiple regulatory inputs that converge on the amino terminus, including phosphorylation events and interactions with regulatory proteins, establishing the amino-terminal region as a control point governing CAX1 activity (Cheng and Hirschi, 2003; Cheng et al., 2004a; Cheng et al., 2004b; Wang et al., 2024).

The biological functions of CAX1 have been interpreted through its role as a calcium transporter (Pittman and Hirschi, 2016b; Pittman and Hirschi, 2024). However, accumulating evidence suggests that this view may be incomplete. Genetic studies have revealed phenotypes that are difficult to explain solely through altered vacuolar calcium sequestration (Yang et al., 2022; Mathew et al., 2024; Sarkar et al., 2024; Rhein et al., 2025). Loss of CAX1 enhances tolerance to anoxia and submergence stress, alters stress-associated transcriptional programs, and modifies cytosolic calcium dynamics during stress recovery (Yang et al., 2022; Mathew et al., 2024). More recently, studies utilizing modular CAX1 constructs demonstrated that transport-deficient amino-terminal fragments can influence anoxia responses despite lacking measurable calcium transport activity (Sarkar et al., 2024; Rhein et al., 2025). Although these observations could be explained by disrupted calcium transport, they also raise the possibility that portions of the CAX1 protein participate in signaling processes independent of transport function.

High-resolution studies have begun to define the molecular changes taking place after phosphorylation-dependent regulation, while high level expression of CAX variants suggest modules of CAX1 have functions independent of transport (Sarkar et al., 2024; Rhein et al., 2025; Wang et al., 2025).

In this review, we examine current understanding of CAX1 structure, regulation, and physiological function, with particular emphasis on the NRR and the amino-terminal half of its psudosymmetrical architecture. We also examine evidence for CAX oligomerization and its potential role as a distinct mechanism of CAX regulation, considering how interactions between CAX proteins may influence transporter activity and physiological function. We summarize evidence supporting established roles in calcium transport while highlighting emerging data suggesting possible transport-independent functions. Finally, we propose a conceptual framework in which the amino-terminal architecture of CAX1 serves as a regulatory module capable of linking membrane transport machinery to environmental stress adaptation. This perspective expands the functional scope of CAX1 and identifies new opportunities for understanding how transport proteins contribute to cellular signaling networks.

## 2 CAX proteins as vacuolar Ca^2+^/H^+^ antiporters

Plant cation/H^+^ exchangers (CAX proteins) are members of the Ca^2+^ Cation Antiporter (CaCA) superfamily and represent a major class of vacuolar Ca^2+^/H^+^ antiporters responsible for calcium sequestration into the vacuole (Maãser et al., 2001; Cai and Lytton, 2004). *Arabidopsis* contains six CAX genes (CAX1–CAX6), which are predominantly localized to the tonoplast and exhibit varying degrees of functional overlap (Mathew et al., 2024). While single-gene mutants often display modest phenotypes under standard growth conditions, higher-order mutants can exhibit severe developmental and physiological defects, highlighting both redundancy and specialization within the family (Cheng et al., 2003; Connorton et al., 2012; Mathew et al., 2024).

Phylogenetic analyses divide plant CAX proteins into two principal groups: Type I-A (CAX1, CAX3, and CAX4) and Type I-B (CAX2, CAX5, and CAX6) (Shigaki and Hirschi, 2006; Shigaki et al., 2006; Emery et al., 2012; Pittman and Hirschi, 2024). Although this classification reflects evolutionary relationships, functional distinctions between the two groups are not always clear (Shigaki and Hirschi, 2006; Emery et al., 2012). CAX3 is the closest homolog of CAX1 and shares substantial sequence similarity, yet the two proteins can display differences in transport characteristics and regulation (Murli et al., 2011). These observations suggest that relatively small sequence differences can produce functional divergence among family members.

**Figure 2:**
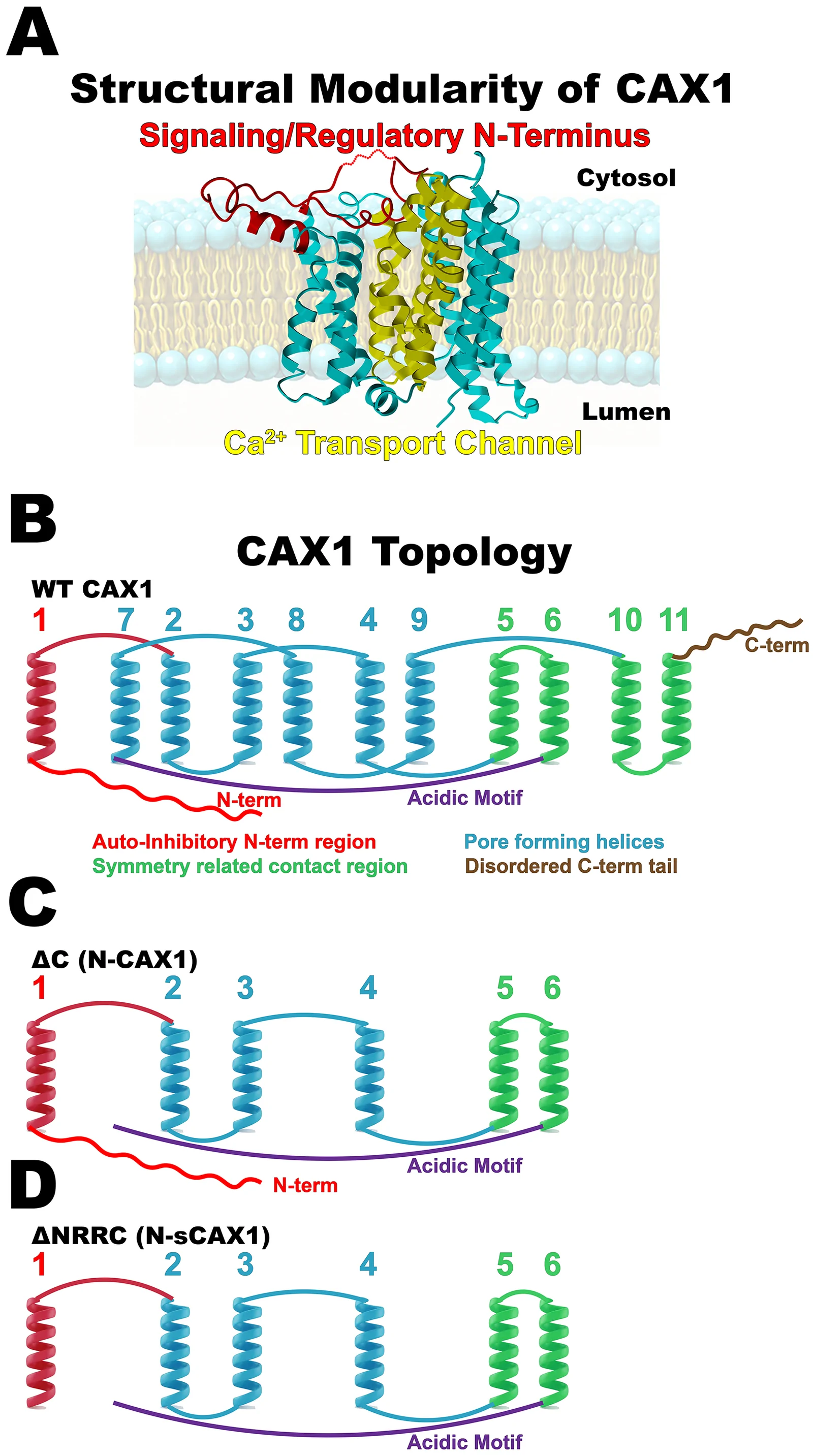
Structural modularity and topology of CAX1. **(A)** Structural model of CAX1 embedded in the tonoplast, highlighting the separation between the membrane-integrated Ca^2+^ transport channel and the cytosolic signaling/regulatory N-terminal region. The transport core spans the membrane, whereas the N-terminus projects toward the cytosol, consistent with a regulatory module positioned to interact with cytosolic signaling factors. **(B)** Topological model of full-length wild-type CAX1. The N-terminal auto-inhibitory region is shown in red, pore-forming helices in cyan, symmetry-related contact regions in green, the acidic motif in purple, and the disordered C-terminal tail in brown. **(C)** Predicted topology of the CAX1-ΔC construct, also referred to as N-CAX1, retaining the N-terminal half of the transporter, including the N-terminal regulatory region, pore-forming helices α1-α6, and acidic motif. **(D)** Predicted topology of the ΔNRRC construct, also referred to as N-sCAX1, in which the N-terminal regulatory region is removed from ΔC, while the remaining N-terminal transport-associated architecture is retained.

Structural analyses indicate that plant CAX proteins share a highly conserved transport architecture (Pittman and Hirschi, 2016a; Pittman and Hirschi, 2016b). Topology for the CaCA-family is often summarized as 10–12 transmembrane helices (TM) forming the central transduction pathway, with plant CAX proteins frequently predicted to contain ~11 helices and a distinctive regulatory amino-terminal element (Pittman and Hirschi, 2001; Emery et al., 2012) (**Figure 2**; **Supplementary Figure 1**). Members of the family are approximately 400 residues in length and are organized into two pseudo-symmetrical halves connected by an acidic linker between helices 6 and 7 (Cai and Lytton, 2004; Pittman and Hirschi, 2016b; Wang et al., 2025) (**Figure 2**). Within this scaffold, conserved α-repeat regions between helices 2 & 7 and between 3 & 8 have been described as forming the cation-binding/exchange pocket (Emery et al., 2012). These are proposed to create a Ca^2+^/H^+^ binding configuration that is compatible with “one-in, one-out” exchange-cycle models (Pittman and Hirschi, 2016b; Demidchik et al., 2018) (**Figure 1**). Similar structural organization is observed throughout the CaCA superfamily, and structures of bacterial and fungal homologs, including yeast VCX1, have provided valuable templates for understanding plant CAX proteins (Nishizawa et al., 2013; Waight et al., 2013; Wu et al., 2013).

Despite strong conservation of the transport core, substantial sequence divergence occurs within cytosolic loops and terminal regions (Manohar et al., 2011; Pittman and Hirschi, 2016a). This pattern suggests that evolutionary diversification of CAX proteins has occurred primarily through modification of regulatory domains while maintaining a conserved transport mechanism (Manohar et al., 2011). The amino-terminal region is particularly notable in plant CAX proteins and represents a major regulatory feature that distinguishes them from many non-plant homologs (Pittman et al., 2002b; Pittman et al., 2002a).

**Figure 3:**
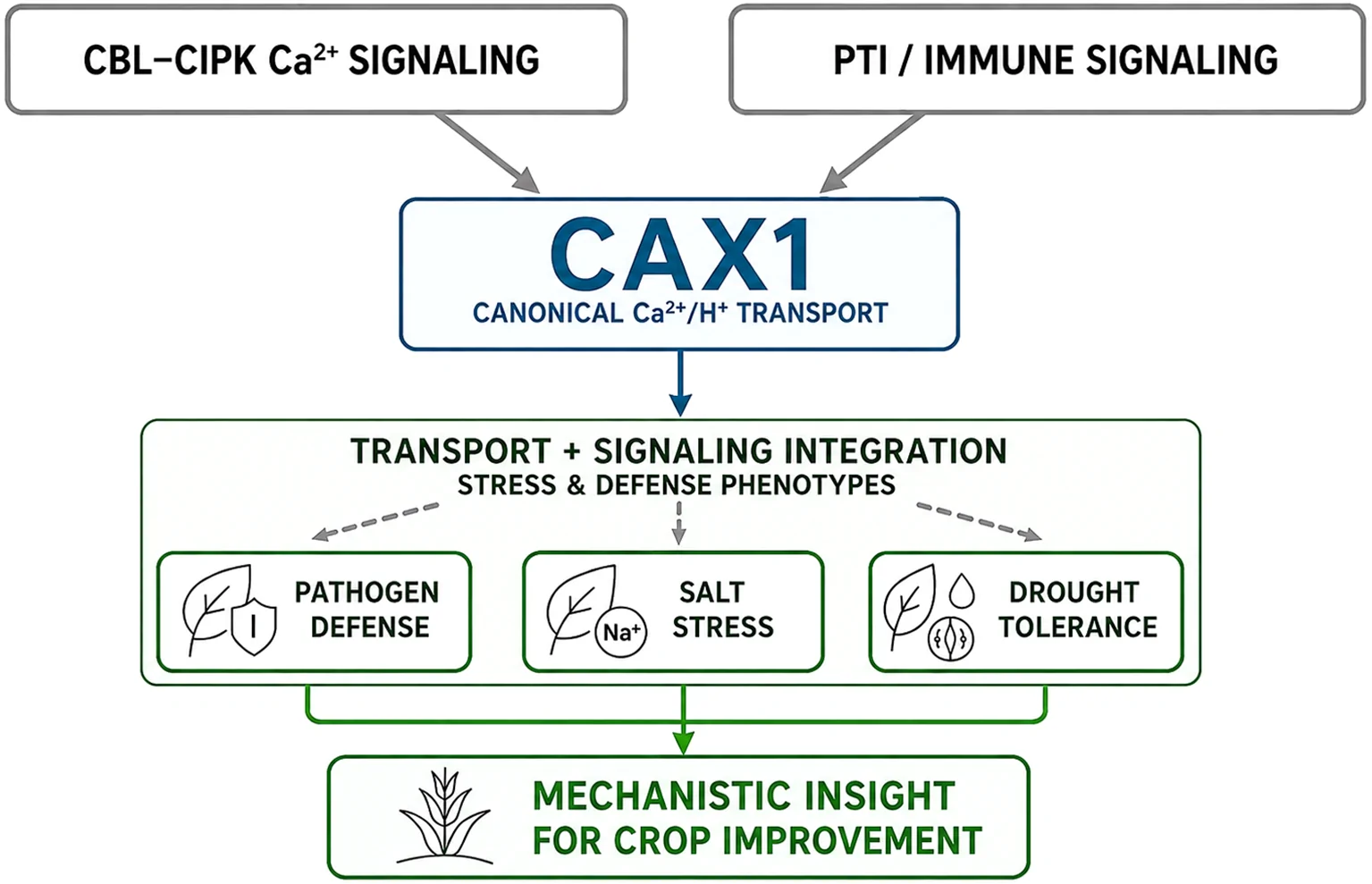
CAX1 as an integrated transport and regulatory node. Conceptual model placing CAX1 at the intersection of Ca^2+^/H^+^ transport, CBL–CIPK calcium signaling, immune signaling, and stress-response pathways. In this model, CAX1 is not limited to vacuolar Ca^2+^ sequestration but may also function as a regulatory hub that connects ion transport with pathogen defense, salt stress, drought tolerance, and broader crop-improvement phenotypes.

Functionally, CAX proteins operate as low-affinity, high-capacity calcium transporters that complement the activities of vacuolar Ca^2+^-ATPases (Pittman et al., 2011; Yang et al., 2022). Whereas Ca^2+^-ATPases function as high-affinity systems that fine-tune cytosolic calcium concentrations, CAX transporters provide bulk calcium sequestration driven by the proton electrochemical gradient (Pittman et al., 2011) (**Figure 1**). Together, these transport systems maintain calcium homeostasis, restore basal calcium levels following signaling events, and shape the calcium dynamics that underlie plant growth and stress responses (Demidchik et al., 2018) (**Figure 3**).

## 3 The amino-terminal regulatory region

A defining feature of plant CAX proteins is the presence of a cytosolic NRR that is absent from many non-plant homologs (Pittman and Hirschi, 2001; Pittman et al., 2002b) (**Supplementary Figure 1**). Early studies established this region as a potent autoinhibitory domain when full-length CAX1 failed to complement vacuolar calcium transport-deficient yeast mutants, whereas deletion of the NRR generated a constitutively active transporter termed short-CAX1 (sCAX1) (Pittman and Hirschi, 2001). These experiments demonstrated that CAX1 is not constitutively active but instead exists in a regulated state controlled by its amino terminus.

Subsequent studies revealed that autoinhibition results from intramolecular interactions between the NRR and adjacent amino-terminal sequences that stabilize an inactive transporter conformation (Pittman et al., 2002a) (**Supplementary Figure 1**). Mutational analyses identified several residues within the NRR that contribute to this inhibitory interaction and established a second regulatory region immediately downstream of the NRR, termed the regulatory-dependent region (RDR) (Pittman et al., 2002a) (**Supplementary Figure 1**). Together, these domains function as a molecular switch that determines whether the transporter remains inactive or transitions into an active state (Pittman et al., 2002a).

Recent structural and biochemical studies have refined this regulatory region model (Wang et al., 2024; Wang et al., 2025). Phosphorylation of a conserved serine cluster located within the RDR promotes transporter activation, while interactions between specific residues in the NRR and RDR stabilize the inactive state (Pittman et al., 2002a; Wang et al., 2024; Wang et al., 2025). Cryo-EM studies further support this regulatory mechanism by demonstrating conformational differences in helix 3 between autoinhibited and activated forms of CAX1 (Wang et al., 2025). Collectively, these findings indicate that the amino terminus functions as a dynamic regulatory module that integrates structural and signaling inputs to control transporter activity.

The physiological importance of this regulation is underscored by the detrimental consequences of constitutive CAX activation (Hirschi, 1999; Zorrilla et al., 2019). Unregulated calcium sequestration can disrupt cellular calcium homeostasis and produce growth defects, indicating that tight control of transporter activity is required for normal plant development and stress responses (Hirschi, 1999; Cheng et al., 2003; Conn et al., 2011; Zorrilla et al., 2019; Zhang et al., 2020).

## 4 Activation of CAX1 by protein interactions and phosphorylation

Autoinhibition of CAX1 is relieved through both protein-protein interactions and phosphorylation-dependent mechanisms that converge on the NRR (Pittman et al., 2002a; Wang et al., 2024). One of the first identified activators was the serine/threonine kinase SOS2 (CIPK24), which interacts with the amino terminus of CAX1 and stimulates transporter activity in yeast complementation assays (Cheng et al., 2004b). Additional regulatory proteins, termed CAX-interacting proteins (CXIPs), also bind the amino terminus and activate CAX1, indicating that this region serves as a platform for regulatory protein interactions (Cheng and Hirschi, 2003; Cheng et al., 2004a).

Phosphorylation has emerged as a second major mechanism controlling CAX1 activity. A conserved serine cluster located within the RDR functions as a possible target for kinase-mediated activation (Pittman et al., 2002a; Wang et al., 2024; Wang et al., 2025). Structural and biochemical studies support a model in which phosphorylation promotes conformational changes that relieve autoinhibition and favor transporter activation (Wang et al., 2024; Wang et al., 2025). Consistent with this model, phosphomimetic regulation through replacement of randomly selected serine/threonine residues within the NRR and RDR yields constitutively active CAX1 mutants that enable transport function studies, they represent an interesting phenomenon that isn’t necessarily physiologically relevant (Pittman et al., 2002a; Wang et al., 2024; Wang et al., 2025). Further kinase-dependent phosphorylation studies are necessary to identify specific serine/threonine residues targeted by kinase activity. This approach would lead to the design of physiologically relevant phosphomimetic CAX1 mutants to better understand the mechanisms through which distinct regulatory pathways control CAX1 activity. We advise that studies of phosphorylated-CAX1 through kinase-associated complexes are necessary and can be explored by Matrix-Assisted Laser Desorption/Ionization Time-of-Flight (MALDI-TOF) mass spectrometry to identify targeted regions and structural studies through cryo-EM/X-ray crystallography to further identify targeted residues and understand structural mechanisms behind activation.

Recent studies have further linked CAX1 to established signaling pathways (Lu et al., 2010; Wang et al., 2024) (**Figure 3**). During pattern-triggered immunity, the kinases Botrytis-induced kinase 1 (BIK1) and PBS1-like protein 1 (PBL1) phosphorylate CAX1 downstream of the Flagellin-sensing 2 protein (FLS2)–Brassinosteroid insensitive 1-associated receptor kinase 1 (BAK1) receptor complex (Wang et al., 2024), while calcium-dependent Calcineurin B-like protein (CBL)–CBL-interacting serine/threonine-protein kinase (CIPK) modules have been implicated in regulating CAX1 activity at the tonoplast (Cheng et al., 2004b; Chaves-Sanjuan et al., 2014; Wang et al., 2024). Together, these findings position the amino-terminal region as a regulatory hub that integrates multiple environmental and developmental cues to control vacuolar calcium transport.

## 5 Expanding the boundaries of the regulatory region

The original model of CAX1 regulation defined the NRR as the first 36 residues of the protein, based on observations that deletion of this segment generated the constitutively active transporter sCAX1 (Pittman and Hirschi, 2001). Subsequent studies identified a RDR immediately downstream of the NRR that contains phosphorylation sites and residues required for activation, suggesting that the amino terminus functions as a coordinated regulatory module rather than as a single inhibitory element (Pittman et al., 2002a).

More recent mutational analyses indicate that the functional boundaries of this regulatory region extend beyond the original 36-residue definition (Han et al., 2022; Wang et al., 2024) (**Supplementary Figure 1**). Truncation studies in *Arabidopsis* CAX1, CAX3, and tomato (*Solanum lycopersicum*) CAX proteins showed that deletion of the first 66 residues (Δ66) resulted in greater transport activity than the classical Δ36 constructs (Han et al., 2022). In contrast, removal of helix α1-A and α1-B abolished transport activity in the tomato CAX1, indicating that this region is required for proper transporter function (Han et al., 2022). These findings suggest that residues beyond the classical NRR contribute to autoinhibition, whereas helix α1-A and α1-B form part of the structural framework necessary for transport.

Recent Cryo-EM and structural modeling studies support this view (Wang et al., 2025). Interactions between residues in the NRR and downstream amino-terminal regions stabilize the autoinhibited state, while phosphorylation-dependent conformational changes within the RDR promote activation (Pittman et al., 2002a; Wang et al., 2024; Wang et al., 2025). Together, these findings support a model in which CAX1 regulation is distributed across a broader amino-terminal module encompassing the NRR, RDR, and helix α1-A and α1-B (Han et al., 2022; Wang et al., 2024). This expanded framework provides a structural basis for understanding how phosphorylation, protein interactions, and environmental signals converge to regulate transporter activity.

## 6 Genetic and physiological evidence beyond transport

**Figure 4:**
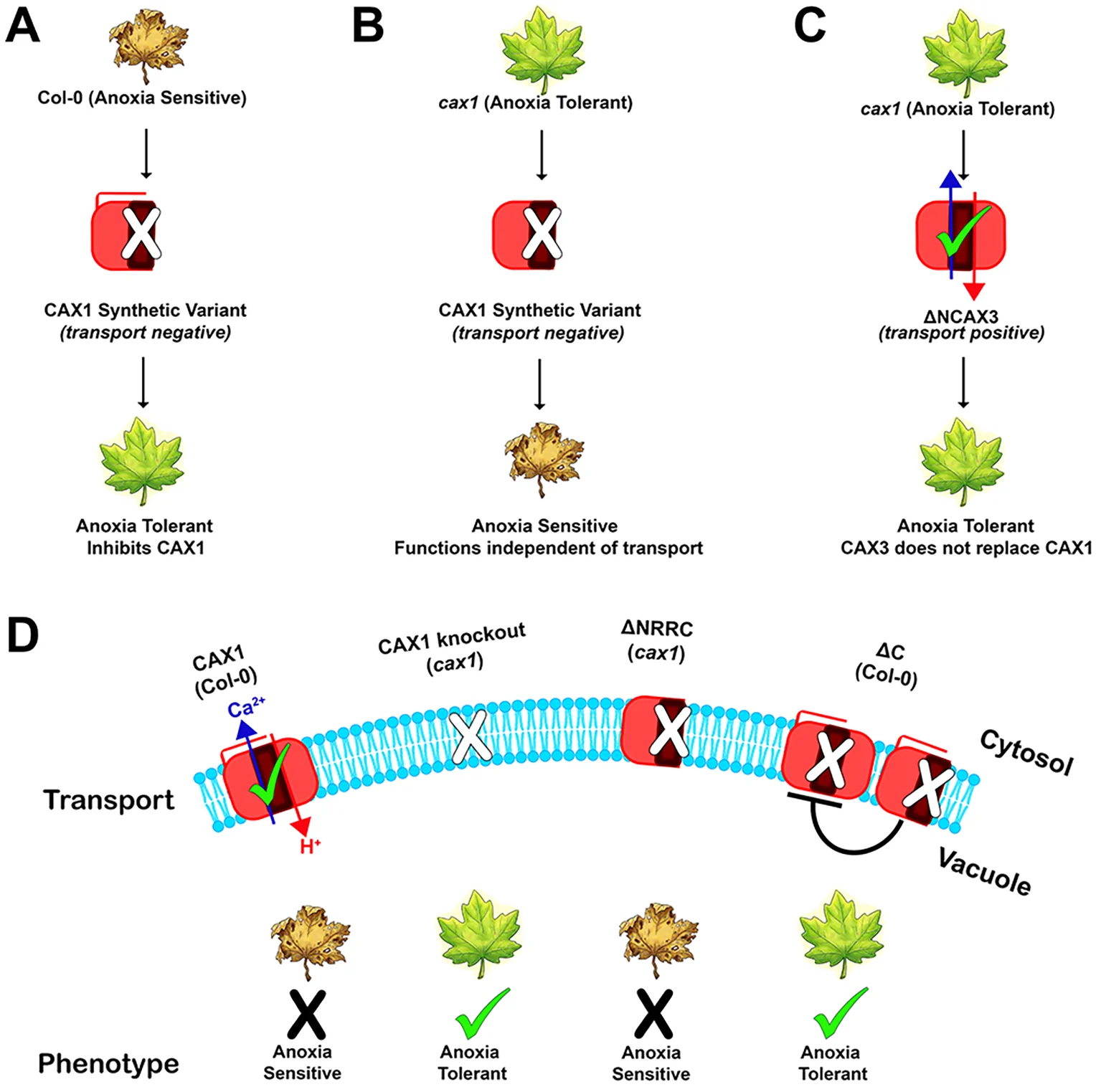
Genetic separation of CAX1 transport activity from anoxia-associated recovery. **(A)** In Col-0 background, which is anoxia sensitive, a transport-negative CAX1 synthetic variant can still support an anoxia-related function, indicating that this output does not require canonical Ca^2+^/H^+^ transport activity. **(B)** Synthetic variants of CAX1 are anoxia tolerant, and high-level expression of a transport-negative CAX1 variant causes anoxia sensitivity. This supports the hypothesis that CAX1 has functions independent of calcium transport. **(C)** High level expression of ΔNCAX3 in *cax1* lines fails to restore anoxia sensitivity. This supports the hypothesis that vacuolar calcium transport alone is insufficient to produce anoxia sensitivity. **(D)** Summary model comparing transport and signaling outputs across CAX1-related genotypes and constructs. Functional CAX1 in Col-0 is transport positive and associated with anoxia sensitivity. Mutants in CAX1 are anoxia tolerant. The ΔNRRC construct lacks transport, but restores sensitivity, supporting the transport-independent anoxia function, whereas high-level expression of the ΔC construct blocks CAX1, conferring anoxic tolerance. These comparisons support a model in which CAX1 contains separable transport and regulatory modules, with the NRR contributing to anoxia-related responses.

Although CAX1 is best known as a vacuolar Ca^2+^/H^+^ antiporter, several observations suggest that its biological functions extend beyond simple calcium sequestration (Yang et al., 2022; Mathew et al., 2024; Sarkar et al., 2024; Rhein et al., 2025). Among the most striking phenotypes is the enhanced tolerance of CAX1 mutants (*cax1*) to low-oxygen stress (Yang et al., 2022; Mathew et al., 2024). Following anoxia or complete submergence, *cax1* mutants exhibit improved recovery, reduced oxidative damage, and lower cell death compared to wild-type plants (Yang et al., 2022). These phenotypes are reversed by reintroduction of CAX1, demonstrating that the stress response is directly linked to CAX1 function (Yang et al., 2022) (**Figure 4**).

Additional phenotypes indicate that CAX1 influences multiple aspects of plant physiology (Pittman and Hirschi, 2024). *cax1* mutants alter vacuolar calcium transport, modify the activity of other tonoplast transport systems, and trigger compensatory changes in expression of related CAX family members (Yang et al., 2022; Mathew et al., 2024). *cax1* mutants also display enhanced tolerance to cold acclimation (Yang et al., 2023), elevated magnesium, and serpentine soil conditions (Bradshaw, 2005), while *cax1*/*cax3* double mutants exhibit severe developmental defects, altered calcium distribution, and impaired growth (Cheng et al., 2005). Conversely, constitutive activation of CAX1 through amino-terminal truncation produces growth defects and calcium-deficiency-like symptoms despite increased total calcium accumulation, highlighting the importance of tight regulatory control (Pittman et al., 2002a; Han et al., 2022).

Beyond these physiological phenotypes, *cax1* mutants exhibit altered cytosolic Ca^2+^ dynamics, distinctive transcriptomic profiles, and extensive proteomic reprogramming (Cheng et al., 2003; Yang et al., 2022). Notably, several hypoxia-responsive genes are expressed in *cax1* plants under aerobic conditions, suggesting that loss of CAX1 partially shifts the cellular stress state prior to oxygen deprivation (Yang et al., 2022) (**Figure 4**). Collectively, these observations are difficult to explain solely through changes in vacuolar calcium storage and instead suggest that CAX1 participates more broadly in the regulation of stress-response pathways.

One intriguing candidate found in *Arabidopsis* linking CAX function to intracellular signaling is CXIP4 (CAX-Interacting Protein 4), which interacts with the N-terminal regulatory region of CAX1 and positively regulates its activity (Cheng et al., 2004a). CXIP4 contains a zinc-knuckle (ZCCHC) motif, that is commonly found in proteins involved in RNA interactions, highlighting its functional role in gene regulation (Aceituno-Valenzuela et al., 2020; Aceituno-Valenzuela et al., 2024). Notably, CXIP4 localizes to both the nucleus and the tonoplast, suggesting functions beyond activation of calcium transport (Cheng et al., 2004a). This dual localization provides a potential mechanism for CAX-mediated signaling. In response to calcium or environmental stress, CXIP4 may re-localize to the tonoplast, where interaction with CAX1 activates transport while initiating a signaling event. CXIP4 could then return to the nucleus to influence gene expression, coupling vacuolar calcium transport with longer-term transcriptional responses. Although speculative, this model offers a testable mechanism by which CAX proteins could function as both transporters and signaling platforms. In wheat (*Triticum aestivum*), a Histidine-rich Calcium Binding Like protein (TaHRC), possesses a NLS (Nuclear Localization Signal) region that allows it to recruit CAX interacting protein 4 (TaCAXIP4) in the nucleus. This suppresses Ca^2+^-mediated immune stress responses in susceptible Fusarium head blight (FHB) plants preventing transport activity of TaCAX1 (Chen et al., 2022). Although in wheat it negatively regulates CAX1 activity, it may be that CBL proteins can initiate recruitment of CXIP4 in order to regulate CAX1 activity in other organisms such as *Arabidopsis*.

## 7 Oligomerization as a mechanism of CAX regulation

In addition to regulation through the NRR, CAX activity can be influenced by interactions between CAX proteins (Zhao et al., 2009; Zhao et al., 2009). CAX1 and CAX3 form both homo- and heteromeric complexes, and co-expression of CAX1 and CAX3 produces transport properties distinct from those observed when either transporter is expressed individually (Hocking et al., 2017). These interactions have been demonstrated using multiple approaches, including co-immunoprecipitation and split-ubiquitin assays, and are associated with altered transport properties in both yeast and plant membranes. Later studies confirmed CAX1–CAX3 complex formation in plant cells and demonstrated that the heteromeric complex displays transport kinetics distinct from either CAX protein alone (Hocking et al., 2017).

Studies using split CAX proteins further suggest that the amino-terminal region participates in these interactions (Zhao et al., 2009). Individually non-functional CAX fragments can associate to reconstitute transport, and the amino-terminal half of sCAX1 can interact with C-terminal portions of either CAX1 or CAX3 to generate functional transporters with altered properties. Thus, oligomerization provides a mechanism through which CAX protein composition and domain interactions can modify transporter function.

This possibility is particularly relevant to recent CAX1 truncation studies. The dominant-negative activity of CAX1 with residues 271 to 463 deleted, termed ΔC (previously ½N-CAX1), a construct containing the NRR and the amino-terminal six-transmembrane module (Sarkar et al., 2024) (**Figure 2**; **Supplementary Figure 1**) is consistent with interference with endogenous CAX1 through an interaction- or assembly-dependent mechanism. Whether the transport-deficient CAX1 with the NRR deleted (residues 1-36) and the C-terminal residues 271-463, termed ΔNRRC (previously ½N-sCAX1), which contains the amino-terminal six-transmembrane module but lacks the NRR (Rhein et al., 2025) (**Figure 2**; **Supplementary Figure 1**) similarly acts through interactions with CAX1, other CAX proteins, or additional partners remains unknown. Nevertheless, the established capacity of CAX proteins and their amino-terminal regions to interact makes oligomerization an important potential means of regulation.

## 8 Evidence for transport-independent regulation by CAX1

The traditional view of CAX1 centers on its role as a vacuolar Ca^2+^/H^+^ antiporter (Hirschi et al., 1996). Under this model, physiological phenotypes associated with CAX1 are largely attributed to altered calcium transport and homeostasis (Manohar et al., 2011; Pittman and Hirschi, 2024). Recent studies, however, have revealed observations that are difficult to explain through transport alone (Bakshi et al., 2023).

Expression of ΔC produces an anoxia-tolerant phenotype resembling that of *cax1* loss-of-function mutants (Sarkar et al., 2024) (**Figure 4**). This dominant-negative effect requires high expression levels and is consistent with inhibition of endogenous CAX1 activity through direct interaction or assembly-dependent mechanisms (Sarkar et al., 2024) (**Supplementary Table 1**).

More compelling evidence comes from expression of ΔNRRC in a *cax1* background, ΔNRRC restores anoxia sensitivity and associated stress phenotypes despite lacking measurable transport activity (Rhein et al., 2025) (**Figure 4**). In contrast, constructs retaining the NRR or expressing only the carboxy-terminal half of the transporter fail to restore sensitivity (Rhein et al., 2025) (**Supplementary Table 1**).

Additional evidence for a CAX1-specific transport function comes from comparisons with CAX3 and ΔNCAX3, a deletion mutant lacking the NRR. Neither version of CAX3 restored anoxia sensitivity to cax1 (**Figure 4**) (**Supplementary Table 1**). These studies demonstrate transport alone is insufficient to restore the anoxia-sensitivity.

Together, these findings suggest that the amino-terminal half of CAX1 contributes to functions that are not readily explained by its measurable Ca^2+^ transport activity. Nonetheless, an indirect contribution of altered Ca^2+^ homeostasis cannot be excluded, as loss or modification of CAX1 transport activity could produce changes in cellular Ca^2+^ dynamics that contribute to the observed phenotypes. Further studies will be required to distinguish between these possibilities and to determine whether the NRR and other amino-terminal regions influence CAX1 function through protein–protein interactions, oligomerization, or other transport-independent mechanisms. Thus, rather than establishing CAX1 as a signaling scaffold, the available evidence raises the possibility that its amino-terminal architecture contributes regulatory functions beyond its established role in Ca^2+^ transport.

## 9 Outstanding questions

Recent studies have expanded our understanding of CAX1 beyond its established role as a vacuolar Ca^2+^/H^+^ antiporter. However, several fundamental questions remain unresolved.

### 9.1 What are the signaling partners of the amino-terminal module?

The proteins responsible for the transport-independent effects of ΔC and ΔNRRC remain unknown, identification of interacting partners that associate during these conditions could be achieved by co-immunoprecipitation, pulldown or proximity labeling assays. While SOS2, CIPK9, CXIPs, BIK1, and related kinases regulate CAX1 activity, identifying proteins that interact with the amino-terminal module may provide the first direct clues to its signaling function.

### 9.2 Is oligomerization required for signaling?

CAX proteins can form homo- and heteromeric complexes, and the dominant-negative behavior of ΔC suggests that protein assembly may influence stress responses. Whether oligomerization is required for transport independent regulation of stress responses requires further studies.

### 9.3 Is regulation independent of transport?

Transport-deficient amino-terminal modules can alter anoxia responses, but complete separation of transport and signaling has not yet been achieved. Definitive evidence will require gene editing approaches that generate separation-of-function mutants that retain one activity while losing the other.

### 9.4 Do other CAX proteins possess transport-independent regulatory functions?

The evidence for transport-independent function has emerged primarily from studies of CAX1. Whether this activity is unique to CAX1 or represents a broader feature of the CAX family remains unknown. Indeed, “signaling” may currently be too strong a term, as the available studies demonstrate biological effects that can be separated from measurable transport but do not yet define a signaling mechanism. Comparative studies of CAX2, CAX3, and other family members will be important for determining whether similar transport-independent regulatory activities occur throughout the CAX family and whether any of these activities can ultimately be defined as signaling.

### 9.5 Can regulation and transport be genetically separated?

Perhaps the most important challenge is determining whether transport and signaling represent mechanistically distinct functions. The ability to genetically uncouple these activities would provide compelling evidence that CAX1 acts as both a transporter and a signaling scaffold, while also creating new opportunities to engineer stress tolerance without disrupting calcium homeostasis.

Resolving these questions will determine whether CAX1 represents a unique example of a transporter-derived signaling protein or whether similar signaling functions are widespread among membrane transporters. Regardless of the outcome, the amino-terminal architecture of CAX1 provides a promising framework for understanding how transport proteins can evolve functions that may extend beyond ion movement alone.

## 10 Concluding remarks

For decades, CAX1 has been viewed as a vacuolar Ca^2+^/H^+^ antiporter that contributes to calcium homeostasis through proton-coupled calcium sequestration. While this framework explains many aspects of CAX1 biology, recent studies suggest a broader regulatory role. The discovery that transport-deficient amino-terminal modules can influence anoxia responses, together with evidence that the amino terminus is regulated through phosphorylation and protein interactions, raises the possibility that CAX1 functions extend beyond ion transport. In this model, the amino-terminal architecture could act as a regulatory module that integrates protein interactions and environmental inputs to influence stress responses independently of measurable calcium transport. Although the underlying mechanisms remain unresolved, this transport-independent regulatory model provides a framework for interpreting observations that are difficult to explain through transport alone.
