# Supplementary material for: CAX1 beyond calcium transport: evidence for a transport-independent regulatory module in plants
Source: Front Plant Sci. 2026 Sep 7;17:1937940. doi: 10.3389/fpls.2026.1937940 (PMC13591097; doi:10.3389/fpls.2026.1937940)
Supplement: Supplementary file 1 [file DataSheet1.docx]

**Supplementary Material**

**Figure S1. Linear domain architecture and contact map of CAX1 constructs.** Linear schematic of full-length CAX1 and deletion constructs showing the amino-terminal regulatory region, pore-forming helices, symmetry-related contact regions, acidic motif, and carboxy-terminal tail. The N-terminal region, including the NRR and RDR, is shown in red and spans the auto-inhibitory regulatory module. Blue segments indicate pore-forming regions and intra-subunit contacts, whereas green segments indicate symmetry-related inter-subunit contacts. The acidic motif is positioned between the N-terminal and C-terminal transport-associated regions, and the C-terminal tail is shown in purple. Construct maps below the full-length protein indicate the retained regions in CAX1-ΔC and CAX1-ΔNRRC, defining the structural boundaries used to separate N-terminal regulatory function from C-terminal transport-associated architecture.

**Table S1.** Functional comparison of CAX1/CAX3 constructs in Arabidopsis anoxia response and yeast Ca²⁺ tolerance assays. Historical construct names are aligned with the revised nomenclature used throughout this manuscript. Constructs were expressed under either the native *CAX1* promoter (pCAX1), corresponding to basal expression, or the cauliflower mosaic virus 35S promoter, corresponding to high expression. Anoxia phenotypes were classified as sensitive or tolerant in the Arabidopsis Col-0 and *cax1* genetic backgrounds. Functional complementation was assessed by growth of the *Saccharomyces cerevisiae* K667 strain on high-calcium medium and is reported as high growth or no growth. ΔN, ΔC, and ΔNRRC denote the corresponding deletion constructs.

| **Old Construct Nomenclature** | **New Construct Nomenclature** | **Promoter** | **Expression** | **Anoxia phenotype in Col-0** | **Anoxia phenotype in *cax1*** | **Yeast K667 on high calcium** |  |
| --- | --- | --- | --- | --- | --- | --- | --- |
| *CAX1* | CAX1 | pCAX1 | Basal | Sensitive | Sensitive | No growth |  |
| *CAX1* | CAX1 | 35S | High | Sensitive | Sensitive | No growth |  |
| *sCAX1* | ∆N-CAX1 | pCAX1 | Basal | Sensitive | Sensitive | High growth |  |
| *sCAX1* | ∆N-CAX1 | 35S | High | Sensitive | Sensitive | High growth |  |
| *½N-CAX1* | ∆C-CAX1 | pCAX1 | Basal | Sensitive | Tolerant | No growth |  |
|  |  |  |  |  |  |  |  |
| *½N-CAX1* | ∆C-CAX1 | 35S | High | Tolerant | Tolerant | No growth |  |
|  |  |  |  |  |  |  |  |
| *½N-sCAX1* | ∆NRRC-CAX1 | pCAX1 | Basal | Sensitive | Tolerant | No growth |  |
|  |  |  |  |  |  |  |  |
| *½N-sCAX1* | ∆NRRC-CAX1 | 35S | High | Sensitive | Sensitive | No growth |  |
|  |  |  |  |  |  |  |  |
| *½C-CAX1* | ∆N-CAX1 | 35S | High | Sensitive | Tolerant | No growth |  |
|  |  |  |  |  |  |  |  |
| *CAX3* | CAX3 | 35S | High | Sensitive | Tolerant | No growth |  |
| *sCAX3* | ∆N-CAX3 | 35S | High | Sensitive | Tolerant | High growth |  |
